# Association between mutations in the *FMR1* gene and ovarian dysfunction in Brazilian patients

**DOI:** 10.5935/1518-0557.20210063

**Published:** 2022

**Authors:** Cinthia Ramos, Maristela Ocampos, Ingrid Tremel Barbato, Viviane Margareth Scantamburlo Niehues, Maria da Graça Bicalho, Renato Nisihara

**Affiliations:** 1 Post Graduate Program in Gynecology and Obstetrics, Federal University of Paraná, Curitiba, Brazil; 2 Neurogene Laboratory of Human Cytogenetics and Molecular Genetics, Florianópolis, Brazil; 3 Department of Genetics, Federal University of Paraná, Curitiba, Brazil; 4 Department of Medicine, Positivo University, Curitiba, Brazil

**Keywords:** fragile X syndrome, mutation, primary ovarian insufficiency

## Abstract

**Objective:**

Our study aimed to identify mutations in the *FMR1* gene in a group of Brazilian women diagnosed with primary ovarian insufficiency (POI).

**Methods:**

This cross-sectional study included patients aged under 40 years with confirmed POI from a convenience sample of patients seen from June 2017 to December 2018 at a University Hospital in Curitiba, Brazil. Genomic DNA was extracted and analyzed using FragilEase(tm) PCR kits (PerkinElmer), a commercially available test that enables the quantification of CGG trinucleotide repeat expansions in the *FMR1* gene.

**Results:**

A total of 52 patients with an average age of 35.8±3.97 years were included. Fifty (96.1%) had normal alleles with 18 to 43 CGG repeats. The most frequent CGG-repeat sizes were 28 and 30. Two patients (3.8%) presented mutations in the FMR1 gene. The first had alleles with 19/97 CGG repeats, was categorized as a premutation carrier for FXS, and had a son with cognitive impairment. The second had alleles with 21/45 CGG repeats and was described as belonging to the gray zone.

**Conclusions:**

In our study, 3.8% of the females with POI had mutations in the FMR1 gene. The most frequent allele sizes were 28 and 30 CGG repeats.

## INTRODUCTION

A variety of disorders have been associated with mutations in the fragile X mental retardation 1 (*FMR1*) gene, including fragile X syndrome (FXS) caused by a full mutation (>200 CGG repeats in the 5′ untranslated region of the *FMR1* gene) leading to absence or deficiency of the *FMR1* protein (FMRP) and premutation (55−200 CGG repeats) disorders characterized by elevations in *FMR1* mRNA to 2−8 times the normal level (Lozano *et al*., 2014). Allingham-Hawkins *et al*. (1999) described a higher incidence of primary ovarian insufficiency (POI) related to the *FMR1* gene and fragile X-associated primary ovarian insufficiency (FXPOI). Expansions in the FMR1 gene may cause intellectual disability and present as FXS (OMIM #300624) in children, a condition more common in boys than in girls. A full mutation (>200 CGG trinucleotide repeats in the *FMR1* gene) leads to hypermethylation, suppression of *FMR1* transcription, and decreased FMR1 protein levels in the brain (Oberlé *et al*., 1991; Verkerk *et al*., 1991). Women with premutation level repeats in this gene (55−200 CGG) are at an increased risk to POI (Allingham-Hawkins et al., 1999; Sullivan et al., 2005). Some authors have suggested that the risk of POI may be highest for repeat sizes between 59 and 99, with lower risk levels described within the range of 100-200 CGG repeats (Sullivan *et al*., 2005).

In addition, a recent review suggested that women with *FMR1* premutation should be informed that they might have lower chances of success with in vitro fertilization (IVF) due to lower number of retrieved oocytes after ovulatory stimulation regimens; interestingly, IVF outcomes are apparently unaffected by *FMR1* repeat lengths smaller than 55 CGG or greater than 200 CGG (Pastore *et al*., 2019).

There is scarce information about FXPOI in the Brazilian population and about women who desire to become pregnant. Our study aimed to identify the number of CGG repeats in the *FMR1* gene in a group of women diagnosed with POI. In addition, we aimed to evaluate the allele frequency of the study population and provide guidance and genetic counseling to women diagnosed with *FMR1* gene mutations.

## MATERIAL AND METHODS

### Ethics Review Committee Approval

The Research Ethics Review Committee of the Federal University of Paraná, Brazil, approved this study and assigned it certificate number CAAE 65993417.0.0000.0096. All included participants signed informed consent terms. No external funding was granted and none of the authors had conflicts of interest to declare. All procedures involving participants were performed in accordance with the ethics standards of the Institution's and/or the National Research Committee and in full compliance with the 1964 Helsinki Declaration and its later amendments and comparable ethics standards.

### Sample and data collection

The study included patients aged under 40 years of age; the diagnosis of POI was confirmed with two measurements of follicle-stimulating hormone (FSH) levels >25 IU/L taken within a minimum interval of four weeks, and oligo or amenorrhea for at least four months (ESHRE, 2016). Ovarian reserve was assessed based on antral follicle counts performed during ultrasound examination and serum levels of FSH, estradiol, and anti-Müllerian hormone (AMH) (Practice Committee of the American Society for Reproductive Medicine, 2015). The patients were followed up at the Gynecology Clinic of the University Hospital. Ours was a convenience sample that included all patients that came for regular appointments from June 2017 to December 2018 who agreed to join the study. Women undergoing chemotherapy during the study period, subjects with autoimmune diseases, and individuals with Turner syndrome were excluded.

### Sample DNA analysis

Samples of venous blood from the included patients were collected in anticoagulant tubes with EDTA. Genomic DNA was extracted from 300 µl of whole blood according to the Wizard^®^ Genomic DNA Purification Kit extraction protocol (Promega Corporation, Madison, WI, USA). DNA was quantified using a NanoDrop® Spectrophotometer ND-1000 instrument, diluted to a final concentration of 25−50 ng/µl, and stored at −20 °C for later use in polymerase chain reaction (PCR) and further analysis with FragilEase(tm) PCR kits (PerkinElmer, Turku, Finland). The FragilEase PCR assay is a commercially available test that enables the quantification of CGG trinucleotide repeat expansions in the *FMR1* gene. Initial PCR screening was performed on all 52 samples. PCR reactions were conducted with primers C (5´GCTCAGCTCCGTTTCGGTTTCACTTCCGGT3´) and F (5´AGCCCCGCACTTCCACCACCAGCTCCTCCA 3´) as described by (Fu *et al*., 1991). FragilEase(tm) PCR analysis was performed on samples with PCR results that showed only a single full-size allele or an allele greater than 40 CGG repeats. FragilEase™ PCR products underwent Capillary Electrophoresis using an ABI 3130 Genetic Analyzer (Applied Biosystems, Foster City, CA) with 50-cm polymer capillaries and POP-7 polymer (Applied Biosystems).

### Patient and Public Involvement

This study was performed without patient involvement. The patients were not invited to comment on the study design and were not consulted to develop patient relevant outcomes or interpret the results. The patients were not invited to contribute in the writing or editing of this manuscript for readability or accuracy.

## RESULTS

The 52 patients included in the study were seen at the infertility clinic and were diagnosed with POI. Participant mean age was 35.8±3.97 years. Seventeen patients (32.7%) were aged less than 35 years and 35 (67.3%) were aged between 35 and 40 years of age.

Most of the patients (88.4%) were Caucasians, 6.9% were of Arab descent, and 4.7% were of African descent. In terms of familial history, 25% had another relative with POI, 25% reported having a relative with cognitive impairment, and 9.6% had a relative with neurodegenerative disease.

**Figure 1:**
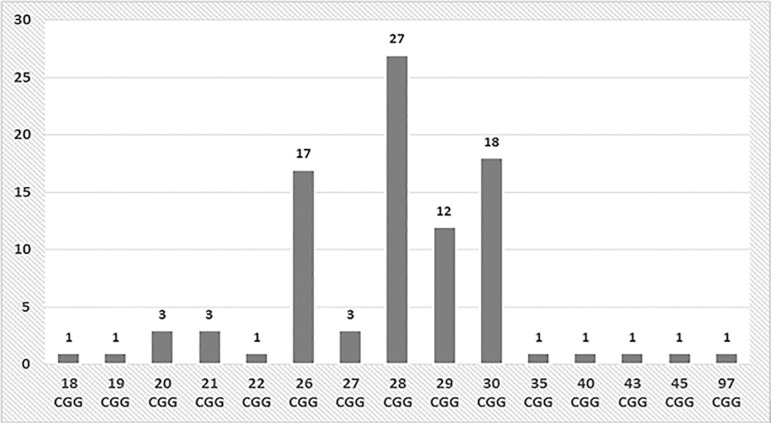
Allele distribution in patients with premature ovarian failure (n=52).

Fifty patients (96.1%) had normal alleles with 18 to 43 CGG repeats. The most frequent allele sizes found in the study were 28 and 30 CGG repeats, as shown in Figure 1. Two patients (3.8%) had mutations in the *FMR1* gene. One (1.9%) had alleles with 19/97 CGG repeats and was categorized as a premutation carrier for FXS. And the other had alleles with 21/45 CGG repeats and was categorized as belonging to the gray zone. In the first case, in which PCR screening detected an allele above 40 CGG repeats, the sample was tested with FragilEase(tm) PCR, which confirmed an allele in the premutation range. When interviewed, the patient (40 years old) did not mention a family history of POI or neurodegenerative disease. She reported having European ancestry and having two children, a boy and a girl, drawing attention to the fact that the boy had a cognitive impairment. The girl did not present with signs suggestive of FXS. In the second case, PCR screening detected an allele above 40 CGG repeats, and the FragilEase(tm) test confirmed an allele in the middle range (21/45 CGG repeats). In the interview, the patient reported having European ancestry and no children; she mentioned a cousin with neurodegenerative disease.

## DISCUSSION

Molecular testing for FXS has a dual role in patients with POI. The first is to determine the probable cause of ovarian insufficiency and identify carriers of mutations in the *FMR1* gene (Streuli *et al*., 2009). The second is to identify FXS premutation alleles so that the carrier may receive genetic counseling about the potential impacts of the disease to the patient and her family. Women with this condition face physical and emotional challenges in their lives. In addition, chronic hypoestrogenism may result in impaired bone health and increased cardiovascular risk (Hoyos & Thakur, 2017). Neuropsychiatric issues include the risk of developing fragile X-associated tremor/ataxia syndrome, neuropathy, musculoskeletal problems, increased prevalence of anxiety, depression, sleep disorders, increased risk of postpartum depression, in addition to the stress inherent to raising a child with FXS (Hoyos & Thakur, 2017). Therefore, women with an FXS premutation benefit greatly from the support of healthcare teams. Awareness of these risks and correlations with various manifestations may assist in early diagnosis and coordination of care for these women and their families.

Regarding the laboratory method used in diagnosis of premutations, it is currently recommended that testing for FXS be performed in people with DI, autism, developmental delay, family members with undiagnosed FXS or DI, cerebellar ataxia, and women with infertility (Monaghan *et al*., 2013). Many laboratories use PCR to determine the number of CGG repeats. However, this technique has its limitations, since it cannot differentiate homozygous alleles in women and rarely amplifies alleles larger than 100−150 CGG repeats (Filipovic-Sadic *et al*., 2010). When there is a need for diagnostic complementation, Southern blotting (SB) - the gold standard for FXS diagnosis - is used, since it detects alleles above 150 CGG repetitions, mosaicism, and methylation patterns of the *FMR1* gene. However, this is a costly, time-consuming method that requires a large amount of genomic DNA (Rajan-Babu & Chong, 2016). In recent years, commercial PCR-based kits have been developed to simplify the workflow in diagnosing FXS with the added capability of detecting mosaicism, identifying homozygous women, and accurately quantifying the number of CGG repeats (Filipovic-Sadic *et al*., 2010). In our country, the main limitation concerning the use of FXS commercial diagnostic kits is their high cost when compared with other PCR-based methodologies. For this reason, PCR is still used as a first-line test, and if there is a need for diagnostic complementation, *FMR1* kits are used as a second-line test because they are more practical and less laborious than SB (Macpherson & Murray, 2016).

Several studies have shown that the frequency of premutation in women with POI is variable. In China, for example, the reported frequency is 0.7%, versus 3.3% in Iran, 4.8% in Slovenia, and 3.0% in Italy (Cheng *et al*., 2017; Asadi *et al*., 2018; Gersak *et al*., 2003; Marozzi *et al*., 2000). Our study found two participants (3.8%) with mutations in the *FMR1* gene, one with a premutation allele and another with an intermediate allele. Global guidelines emphasize the need to investigate premutation for FXS in individuals with a family history or suggestive clinical signs (Monaghan *et al*., 2013). Our findings showed that 3.8% of the patients in the study had mutations in the *FMR1* gene and, in our opinion, the clinical repercussions for patients and relatives with premutation justify performing the examination.

Our study has limitations. One is the small size of our population, which does not allow for statistical analysis. The use of family data may also introduce some recall bias.

To conclude, we identified two (3.8%) patients with mutations in the *FMR1* gene in a group of women with POI. The most frequent allele sizes found were 28 and 30 CGG repeats. Patients identified with the mutation received genetic counseling.

## References

[r1] Allingham-Hawkins DJ, Babul-Hirji R, Chitayat D, Holden JJ, Yang KT, Lee C, Hudson R, Gorwill H, Nolin SL, Glicksman A, Jenkins EC, Brown WT, Howard-Peebles PN, Becchi C, Cummings E, Fallon L, Seitz S, Black SH, Vianna-Morgante AM, Costa SS (1999). Fragile X premutation is a significant risk factor for premature ovarian failure: the International Collaborative POF in Fragile X study--preliminary data. Am J Med Genet.

[r2] Asadi R, Omrani MD, Ghaedi H, Mirfakhraie R, Azargashb E, Habibi M, Pouresmaeili F (2018). Premutations of FMR1 CGG repeats are not related to idiopathic premature ovarian failure in Iranian patients: A case control study. Gene.

[r3] Cheng YKY, Lin CSW, Kwok YKY, Chan YM, Lau TK, Leung TY, Choy KW (2017). Identification of fragile X pre-mutation carriers in the Chinese obstetric population using a robust FMR1 polymerase chain reaction assay: implications for screening and prenatal diagnosis. Hong Kong Med J.

[r4] Webber L, Davies M, Anderson R, Bartlett J, Braat D, Cartwright B, Cifkova R, de Muinck Keizer-Schrama S, Hogervorst E, Janse F, Liao L, Vlaisavljevic V, Zillikens C, Vermeulen N, European Society for Human Reproduction and Embryology (ESHRE) Guideline Group on POI (2016). ESHRE Guideline: management of women with premature ovarian insufficiency. Hum Reprod.

[r5] Filipovic-Sadic S, Sah S, Chen L, Krosting J, Sekinger E, Zhang W, Hagerman PJ, Stenzel TT, Hadd AG, Latham GJ, Tassone F (2010). A novel FMR1 PCR method for the routine detection of low abundance expanded alleles and full mutations in fragile X syndrome. Clin Chem.

[r6] Fu YH, Kuhl DP, Pizzuti A, Pieretti M, Sutcliffe JS, Richards S, Verkerk AJ, Holden JJ, Fenwick RG Jr, Warren ST, Oostra BA, Nelson DL, Caskey CT (1991). Variation of the CGG repeat at the fragile X site results in genetic instability: resolution of the Sherman paradox. Cell.

[r7] Gersak K, Meden-Vrtovec H, Peterlin B (2003). Fragile X premutation in women with sporadic premature ovarian failure in Slovenia. Hum Reprod.

[r8] Hoyos LR, Thakur M (2017). Fragile X premutation in women: recognizing the health challenges beyond primary ovarian insufficiency. J Assist Reprod Genet.

[r9] Lozano R, Rosero CA, Hagerman RJ (2014). Fragile X spectrum disorders. Intrac Rare Dis Res.

[r10] Macpherson JN, Murray A (2016). Development of Genetic Testing for Fragile X Syndrome and Associated Disorders, and Estimates of the Prevalence of FMR1 Expansion Mutations. Genes (Basel).

[r11] Marozzi A, Vegetti W, Manfredini E, Tibiletti MG, Testa G, Crosignani PG, Ginelli E, Meneveri R, Dalprà L (2000). Association between idiopathic premature ovarian failure and fragile X premutation. Hum Reprod.

[r12] Monaghan KG, Lyon E, Spector EB, American College of Medical Genetics and Genomics (2013). ACMG Standards and Guidelines for fragile X testing: a revision to the disease-specific supplements to the Standards and Guidelines for Clinical Genetics Laboratories of the American College of Medical Genetics and Genomics. Genet Med.

[r13] Oberlé I, Rousseau F, Heitz D, Kretz C, Devys D, Hanauer A, Boué J, Bertheas MF, Mandel JL (1991). Instability of a 550-base pair DNA segment and abnormal methylation in fragile X syndrome. Science.

[r14] Pastore LM, Christianson MS, McGuinness B, Vaught KC, Maher JY, Kearns WG (2019). Does theFMR1 gene affect IVF success?. Reprod BioMed Online.

[r15] Practice Committee of the American Society for Reproductive Medicine (2015). Testing and interpreting measures of ovarian reserve: a committee opinion. Fertil Steril.

[r16] Rajan-Babu IS, Chong SS (2016). Molecular Correlates and Recent Advancements in the Diagnosis and Screening of FMR1-Related Disorders. Genes (Basel).

[r17] Streuli I, Fraisse T, Ibecheole V, Moix I, Morris MA, de Ziegler D (2009). Intermediate and premutation FMR1 alleles in women with occult primary ovarian insufficiency. Fertil Steril.

[r18] Sullivan AK, Marcus M, Epstein MP, Allen EG, Anido AE, Paquin JJ, Yadav-Shah M, Sherman SL (2005). Association of FMR1 repeat size with ovarian dysfunction. Hum Reprod.

[r19] Verkerk AJ, Pieretti M, Sutcliffe JS, Fu YH, Kuhl DP, Pizzuti A, Reiner O, Richards S, Victoria MF, Zhang FP, Eussen BE, Ommen GJB, Blonden LAJ, Riggins GJ, Chastain JL, Kunst CB, Galjaard H, Caskey CT, Nelson DL, Oostra BA (1991). Identification of a gene (FMR-1) containing a CGG repeat coincident with a breakpoint cluster region exhibiting length variation in fragile X syndrome. Cell.

